# Phytochemical Characterization and Evaluation of Biological Activities of Egyptian Carob Pods (*Ceratonia siliqua* L.) Aqueous Extract: In Vitro Study

**DOI:** 10.3390/plants10122626

**Published:** 2021-11-29

**Authors:** Wael Sobhy Darwish, Abada El Sayed Khadr, Maher Abd El Naby Kamel, Mabrouk A. Abd Eldaim, Ibrahim El Tantawy El Sayed, Hamed Mohamed Abdel-Bary, Sami Ullah, Doaa Ahmed Ghareeb

**Affiliations:** 1 Chemistry Department, Faculty of Science, Menoufia University, Menoufia 32511, Egypt; wael_science@yahoo.com (W.S.D.); d.abada_khadr@yahoo.com (A.E.S.K.); ibrahimtantawy@yahoo.co.uk (I.E.T.E.S.); hamed.abdelbari@science.menofia.edu.eg (H.M.A.-B.); 2 Biochemistry Department, Medical Research Institute, Alexandria University, Alexandria 21561, Egypt; maher.kamel@alexu.edu.eg; 3 Department of Biochemistry and Chemistry of Nutrition, Faculty of Veterinary Medicine, Menoufia University, Menoufia 32511, Egypt; 4 Research Center for Advanced Materials Science (RCAMS), King Khalid University, P.O. Box 9004, Abha 61413, Saudi Arabia; samiali@kku.edu.sa; 5 Department of Chemistry, College of Science, King Khalid University, P.O. Box 9004, Abha 61413, Saudi Arabia; 6 Bio-Screening and Preclinical Trial Lab, Biochemistry Department, Faculty of Science, Alexandria University, Alexandria 21511, Egypt; d.ghareeb@alexu.edu.eg

**Keywords:** *Ceratonia siliqua*, carob, flavonoids, polyphenols, antihemolytic, antimicrobial

## Abstract

*Ceratonia siliqua* (Carob) is an evergreen Mediterranean tree, and carob pods are potentially nutritive and have medicinal value. The present study was carried out to estimate the possible biological activities of phytochemical-characterized carob pod aqueous extract (CPAE). The phytochemical contents of CPAE were determined by using colorimetric methods and HPLC. In addition, the free radical scavenging properties and anti-diabetic, anti-hemolytic, and antimicrobial activities were estimated by using standardized in vitro protocols. The phytochemical analysis revealed that CPAE was rich in polyphenols, flavonoids, and alkaloids, where it contained a significant amount of gallic acid, catechin, and protocatechuic acid. Furthermore, CPAE exhibited strong antioxidant activity where it prevented the formation of 2, 2-Diphenyl-1-picryl hydrazyl, hydroxyl, and nitric oxide free radicals. Additionally, it had a potent inhibitory effect against digestive enzymes (amylase, maltase, sucrase, and lactase). Moreover, CPAE exhibited anti-*Staph aureus*, anti-*Escherichia coli*, anti-*Candida albicans*, and anti-herpes simplex type I virus (HSV-I). Finally, CPAE protected the erythrocyte membrane from hypotonic solution-induced hemolysis. Altogether, CPAE could be regarded as an interesting source of biologically active antioxidant, anti-diabetic, and antimicrobial preparation for a potential application in pharmaceutical and food supplement fields.

## 1. Introduction

Plants are of great interest in drug discovery and are the main source of our modern medicine. About 25% of modern medicines are derived from a plant origin and only 5–15% of plants are being investigated for their medicinal use [1]. Today, medicinal herbs and functional foods are being widely studied resulting in lucrative therapeutic potentials [2]. Where the secondary metabolites, which possessed antioxidant properties can fight several pathological disorders such as cancer, heart disease, hypertension, dementia, and stroke [3]. However, chemotherapies have many adverse effects; several anti-diabetic drugs such as Rosiglitazone and Alogliptin can have severe complications. Therefore, scientists have tried to find naturally occurring anti-diabetic compounds as they have low toxic effects [4]. In addition, the WHO has stated that if there is no action taken to develop/find new antibiotic agents, the deaths due to infection resistance will be greater than cancer [5]. Bacteria develop resistance against antibiotics due to the presence of multi-drug resistance (MDR) pumps which are predominant in *Staphylococcus aureus*, *Pseudomonas aeruginosa*, *Escherichia coli*, etc. Therefore, researchers are trying to find MDR inhibitors, especially those derived from medicinal plants, due to their diversity to increase the efficacy of antibiotics [6].

*Ceratonia siliqua* L., Carob, is an evergreen tree that belongs to the *Leguminosae* family. It is widely cultivated in most Mediterranean basin countries [7]. The carob fruit (bean) is called a pod and is composed of two parts: the seeds (10%) and the pulp (90%). The pulp consists of two parts: a leathery outer layer (pericarp) and a softer inner area (mesocarp) which contain the seeds [8,9]. Carob pulp contains a wide range of biologically active compounds, including polyphenols, sugars, cyclitols, amino acids, fibers, and minerals, whereas carob seeds contain gum, polyphenols, and proteins [10]. Due to its chemical composition, carob and its ethanolic extract have a strong antioxidant activity and possess several valuable therapeutic functions, such as lipid-lowering [11], anti-proliferative, anti-cardiovascular [12], and nephroprotective properties [13]. In addition, carob has exhibited significant pharmacological activities in the digestive tract including antidiarrheal, antibacterial, anti-ulcer, and anti-inflammatory actions, and possesses a laxative effect on gastrointestinal propulsion [14].

Despite carob’s importance in functional food development, most Arab countries use carob to make a popular drink consumed mainly in the month of Ramadan. Carob is also used in the preparation of special traditional types of Arabic confectionery. Due to the nutritional value of carob powder water extract which is known to contain dietary fiber, tannins, flavonoids, and gallic acid, which account for its antioxidant properties, and due to the lack of information about its therapeutic value, this study aimed to examine the possible biological activities of this easily and cheaply prepared extract of Egyptian carob pods. The most important biological activities that were measured were antimicrobial and antidiabetics, besides anti-inflammatory and antioxidants properties, even in the presence of high sugar and fiber content in water extract due to the extraction method. Moreover, phytochemical compounds were characterized chemically and by HPLC.

## 2. Materials and Methods

### 2.1. Preparation of Carob Pod Aqueous Extract

The mature carob pods were collected from the farms of the City of Scientific Research and Technological Applications (SRTA-City), New Borg El-Arab City, Alexandria, Egypt. The pods were washed with distilled water to remove debris and dried. The seeds were removed from the pods. Then, the pods were ground to a fine powder with a grinder. Two hundred grams of fine dry powder were soaked in one liter of distilled water and boiled for one hour after that the mixture was incubated for 24 h in a shaker incubator. Then, the mixture was filtrated by gauze to remove fiber and insoluble particles. After that, the filtrate was lyophilized to obtain a fine powder (30 g) that was stored at −20 until used.

### 2.2. Phytochemical Analysis of the Carob Extracts

#### 2.2.1. Quantification of the Total Flavonoid Content

The total flavonoid content was determined by using the aluminum chloride colorimetric method [15]. Briefly; 0.1 mL of the CPAE (1 mg/mL in water) or each rutin concentration (2, 4, 6, 8, and 10 mg/mL in distilled water) was mixed with 1.5 mL of distilled water and 750 µL of sodium nitrite solution, and incubated for 6 min at room temperature. Then, a volume of 150 µL of aluminum chloride solution was added, mixed well, and the absorbance was measured immediately at 510 nm using an optima spectrophotometer. The test was carried out in triplicate. The levels of total flavonoid contents were determined as milligram rutin equivalents per g lyophilized powder, and then the data were converted into milligram rutin equivalents 100 mg CPAE.

#### 2.2.2. Quantification of the Total Phenolic Content

Total phenolic content was determined by using Folin- Ciocalteu colorimetric method described by Taga et al. (1984) [16]. Folin-Ciocalteau reagent (100 µL) and 2 mL sodium carbonate were added and mixed well with 100 µL of CPAE (1 mg/1 mL) or each gallic acid (GA) standard; each mixture was incubated at 25 °C for 2 h. The absorbance of the resulting blue color solution was measured at 750 nm using an optima spectrophotometer. The test was carried out in triplicate. The data were converted to milligram gallic acid equivalents (GAE)/100 mg CPAE.

#### 2.2.3. Quantification of the Total Alkaloid Content

Total alkaloid content was determined by using the method developed by Ayoola et al. (2008) [17] which is based on the reaction between alkaloid with bromocresol green (BCG), resulting in a yellow-colored product. This method has the benefits of sensitivity and stability. In brief, 0.1 mL of CPAE was mixed with 2 N HCl, and one milliliter of this solution was transferred to a separatory funnel and washed (three times) using 10 mL chloroform. The pH of this solution was adjusted to neutral with 0.1 N NaOH. Then 5 mL of BCG solution and 5 mL of phosphate buffer were added to this solution. After that, the mixture was shaken and the complex formed was extracted with 1, 2, 3, and 4 mL chloroform using vigorous shaking. The extracts were collected in a 10 mL volumetric flask and diluted to volume with chloroform. The absorbance of the complex in chloroform was measured at 470 nm. Alkaloids (mg/mL) = (Abs − 0.048)/0.021.

#### 2.2.4. High-Performance Liquid Chromatography (HPLC) Analysis of CPAE

A sample (1 g) of CPAE was placed in a quick-fit conical flask, 20 mL of 2M NaOH was added, the flasks were flushed with N2, and the stopper was replaced. The samples were shacked for 4 h at room temperature. The pH was adjusted to 2, with 6 M HCl. The samples were centrifuged at 5000 rpm for 10 min and the supernatant was collected. Phenolic compounds were extracted twice with 50 mL ethyl ether and ethyl acetate 1:1. The organic phase was separated and evaporated at 45 °C and the samples were redissolved in 2 mL of methanol. HPLC analysis was carried out using Agilent Technologies 1100 series liquid chromatograph equipped with an autosampler and a diode-array detector. The analytical column was an Eclipse XDB-C18 (150 × 4.6 µm; 5 µm) with a C18 guard column (Phenomenex, Torrance, CA). The mobile phase consisted of acetonitrile (solvent A) and 2% acetic acid in water (*v*/*v*) (solvent B). The flow rate was kept at 0.8 mL/min for a total run time of 70 min and the gradient program was as follows: 100% B to 85% B in 30 min, 85% B to 50% B in 20 min, 50% B to 0% B in 5 min, and 0% B to 100% B in 5 min. The injection volume was 50 µL and peaks were monitored simultaneously at 280 and 320 nm for the benzoic acid and cinnamic acid derivatives, respectively. All samples were filtered through a 0.45 µm Acrodisc syringe filter, Pall Corporation, USA, (Gelman Laboratory, MI) before injection. Peaks were identified by congruent retention times and UV spectra and compared with those of the standards [18].

#### 2.2.5. Quantification of the Total Carbohydrate Content

The total carbohydrate content was determined using anthrone reagent (Hedge and Hofreiter, 1962) [19]. Briefly; 0.1 mL of the CPAE was hydrolyzed by keeping it for three hours in a boiling water bath with 5 mL of 2.5 N HCl. The solution was neutralized by adding sodium carbonate until effervescence ceased, the volume was completed to 100 mL with distilled water then centrifuged for 5 min and the supernatants were used for analysis. Different aliquots of standard glucose were used (0, 0.2, 0.4, 0.6, 0.8, and 1 mL). The volumes were completed to 1 mL with distilled water, and then 4 mL of anthrone reagent were added and the solutions were heated for eight minutes in a boiling water bath. The green to dark green color was read at 630 nm. The content of carbohydrate present in 0.1 mL of the sample = (mg of glucose/volume of the test sample) × 100.

#### 2.2.6. Quantification of the Total Amino Acid Content

The total amino acids content was determined according to Moor and Stein (1957) [20]. Briefly; 0.1 mL of the CPAE was taken in a 20 mL test tube and then 1 mL of ninhydrin solution was added to it. The tubes were heated for 20 min in a boiling water bath. Then they were cooled, and 5 mL of the ethanol were mixed and incubated at room temperature for 15 min. The optical density was read at 570 nm using a spectrophotometer. The concentration of unknown was calculated from the standard curve.

### 2.3. Bio-Screening Analysis of CPAE

#### 2.3.1. Determination of Anti-Digestive Enzyme Activities

##### Determination of α-Amylase Activity

A volume of 20 µL amylase (1:100 *w*/*v*) was mixed with 5 µL (10 mg/100 mL) of CPAE and 100 µL phosphate buffer (pH = 6.9); then, the mixture was incubated for 45 min at room temperature. After incubation, 20 µL of soluble starch (1:100 *w*/*v*) was added and the mixture was incubated for 20 min. Then, 100 µL of glucose detection reagent was added to the previous mixture and incubated at room temperature for 20 min. Absorbance was measured at 490 nm wavelength. Specific α-Amylase activity is reported as U = μmol of glucose min^−1^ [21].

##### Determination of Maltase, Sucrase and Lactase Activities

The activities of the disaccharidases enzymes were determined as previously described by Dahlqvist, et al. (1974) [22]. A volume of 10 µL of pancreatin (0.1 g/10 mL H_2_O containing drops of NaOH) was mixed with 5 µL of CPAE (10 mg/100 mL) and 50 µL phosphate buffer (pH 7.4), and the mixture was incubated for 45 min at room temperature, then 10 µL of maltose, sucrose, or lactose (1 g%) as substrate was added and the mixture was left for 20 min at room temperature. The reaction was stopped by thermal denaturation, and 100 µL of glucose detection reagent was added to the previous mixture and incubated for 20 min at room temperature. Absorbance was measured at 450 nm wavelength. The specific activity of the enzyme was reported as U = µmol of glucose min^−1^.

#### 2.3.2. Determination of 2,2-Diphenyl-1-picryl hydrazyl (DPPH) Radical Scavenging Activity

The DPPH radical scavenging activity was determined according to the method described by Blios, (1958) [23]. Briefly, 100 µL of DPPH was mixed with 100 µL of different concentrations of CPAE, 100 µL standard (Ascorbic acid) or 100 µL DEMSO (control). The reaction mixture was thoroughly vortexed and left in the dark at room temperature for 30 min. The absorbance of the mixture was measured spectrophotometrically at 517 nm. The ability of CPAE to scavenge DPPH radical was calculated by the equation:

$$\mathrm{DPPH}\;\mathrm{radical}\;\mathrm{scavenging}\;\mathrm{activity}=\;\left(A\;\mathrm{control}-A\;\mathrm{sample}\;\right)/\left(A\;\mathrm{control}\;\right)\;\times \;100$$

#### 2.3.3. Determination of Hydroxyl Radical Scavenging Activity

This assayed as described by Elizabeth and Rao (1990). The assay is based on the degradation of 2-deoxy ribose by hydroxyl radical generated by the Fe^3+^ Ascorbate –EDTA –H_2_O_2_ system (Fenton reaction). The degradable 2-deoxy ribose was condensed with thiobarbituric acid (TBA) and then measured as TBARS.

#### 2.3.4. Determination of Ferric Reducing Antioxidant Power (FRAP)

One milliliter of CPAE with different concentrations (100, 75, 50, 12.5, and 6.25 mg/mL) was mixed with 1 mL of sodium phosphate buffer (pH 6.6) and 1 mL of potassium ferricyanide. The mixtures were incubated for 20 min at 50 °C, followed by the addition of 1 mL of trichloroacetic acid (TCA 10%), then centrifuged for 10 min at 2000 rpm. One mililiter of the supernatant was added to 1 mL of deionized water and 200 μL of FeCl_3_. The blank was prepared in the same manner as the sample, except that potassium ferricyanide was replaced by distilled water, and the mixture was incubated for 30 min at room temperature. The absorbance of the reaction mixture was measured at 700 nm wavelength (Sutharsingh, et al., 2011) [24].

#### 2.3.5. Determination of Nitric Oxide (NO) Radical Scavenging Activity

Briefly, 400 μL of sodium nitroprusside and 100 µL of phosphate buffer were mixed with 100 μL of CPAE, standard, or DMSO (control). This reaction mixture was incubated for 2.5 h in a dark place at 25 °C. After incubation, 20 µL of Griess reagent was added to the previous mixture and allowed to stand for 30 min at room temperature. The absorbance was measured at 540 nm wavelength (Garratt, 1964) [25].

#### 2.3.6. Hemolytic Activity Assay

The test was performed according to the method described by Farias et al. (2013) [11], with some modifications. Firstly, a serial dilution of CPAE was prepared with 0.9% NaCl ranging from1000 to 1.9 μg⋅mL^−1^ and reserved. Then, 100 μL of a 1% red blood cell (A, B, and O human blood types) suspension was added to a new microtube containing 900 μL of CPAE dilution, then incubated at 37∘C for 1h. After that, the tubes were centrifuged at 3000× *g* for 5min. The supernatant (200 μL) was placed in a 96-well plate and read at 540 nm using a microplate reader. The cell suspensions of each human blood type (100 μL) were mixed with distilled water or 0.9% NaCl (900 μL) to obtain the absorbance for 100 and 0% of cell lysis, respectively. The percentage of hemolysis was calculated as follows: % hemolysis = Abs test/Abs *pc* × 100, where Abs test = Abs540 of the 1% cell suspension treated with sample test and Abs *pc* = Abs540 of the 1% cell suspension treated with distilled water.

### 2.4. Antimicrobial Activity

#### 2.4.1. Antibacterial and Antifungal Method

The antimicrobial activities of the CPAE were studied on Mueller–Hinton agar plates by Agar diffusion technique against Gram-positive (*Bacillus subtilis* (ATCC 6051) and *Staphylococcus aureus* (ATCC 6538) and Gram-negative (*Escherichia coli* (ATCC 8739) and *Pseudomonas aeruginosa* (ATCC 90274)) pathogenic bacterial strains beside fungi (*Aspergillus falvus* (ATCC 9643) as well as yeast *Candida albicans* (ATCC 10221). Gentamicin (10 mg/mL) was used standardly as a positive control. Mueller–Hinton agar plates seeded with 1.8 × 10^8^ cfu/mL (0.5 OD^600^) of the test bacteria. Following 24 h of incubation at 37 °C, plates were examined for the presence of inhibition zones. The inhibition zones surrounding the wells were measured (mm) [26].

#### 2.4.2. Antiviral Method

The Vero cells (ATCC CCL-88) were propagated in Dulbecco’s modified Eagle’s medium (DMEM) supplemented with 10% heat-inactivated fetal bovine serum (FBS), 1% L-glutamine, HEPES buffer, and 50 mg/mL gentamicin. All cells were maintained at 37 °C in a humidified atmosphere with 5% CO_2_ and were subcultured two times a week [27].

##### Evaluation of the Maximum Non-Toxic Concentration (MNTC) of CPAE on Vero Cells

This assay was selected to show the maximum non-toxic concentration of CPAE in susceptible mammalian cells (Hu, et al. 2008). The Vero cells (ATC CCL-88) were propagated in a growth medium (Dulbecco’s modified Eagle’s medium (DMEM) supplemented with 10% heat-inactivated fetal bovine serum (FBS), 1% L-glutamine, HEPES buffer, and 50 mg/mL gentamicin). All cells were maintained at 37 °C in a humidified atmosphere with 5% CO_2_. Growth medium was decanted from 96-well microtiter plates after the formation of the confluent sheet of Vero cell, and the cell monolayer was washed twice with wash media PBS. Different concentrations of CPAE were prepared in DMEM. A volume of 100 μL of each dilution and 100 μL of culture medium (DMEM supplemented with 2% FBS, 1% L-glutamine, HEPES buffer, and 50 mg/mL gentamicin) were added to cells then the plate was incubated at 37 °C for 48 h. After that, 20 μL of MTT solution (5 mg/mL in PBS) (Bio Basic Canada Inc, Toronto, Canada) was added to each well, then the plate was incubated at (37 °C, 5% CO_2_) for 4 h. The media was discarded and the formed formazan (MTT metabolic product) was suspended in 200 µL DMSO. The optical density was read at 560 nm wavelength using an ELISA reader (TECAN Inc., Chapel Hill, NC, USA) and the background was subtracted at 620 nm wavelength [27].

##### Evaluation of the Antiviral Activity of CPAE Using MNTC

In brief, a monolayer of 1 × 10^4^ (Vero cells/well) was placed in a 96-well plate. The plate was incubated at (37 °C, 5% CO_2_) overnight. Then, 100 µL herpes simplex type I virus (HSV-I)/CPAE suspension which was incubated for one hour was added to cells and incubated for 24 h at (37 °C, 5% CO_2_). Then, the cell viability was determined by MTT assay.

### 2.5. Statistical Analysis

All tests were analyzed in triplicate of three independent measurements and the results were expressed as mean values ± standard deviation (SD). The statistical difference was measured with a t-test when it was applicable, at *p* < 0.05, by using SPSS v. 18.0 software.

## 3. Results

### 3.1. The Phytochemical Ingredients of the CPAE

The phytochemical analysis revealed that CPAE contained many active compounds including total phenolics, flavonoids, carbohydrates, amino, acids, and alkaloids (Table 1).

**Figure 1:**
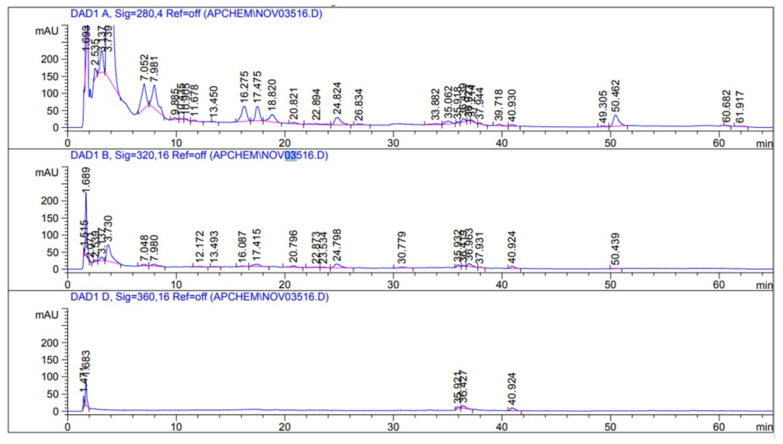
High-Performance Liquid Chromatography (HPLC) analysis of CPAE.

Moreover, HPLC analyses showed that CPAE contained a high content of gallic acid followed by catechin, protocatechuic acid, and cinnamic acid, while it also contained a low concentration of *p*-coumaric acid, rutin, gentisic acid, p-hydroxybenzoic acid, vanillic acid, and ferulic (Figure 1 and Table 2).

### 3.2. The Antioxidant Effect of CPAE

The antioxidant effect of CPAE was measured in vitro. The scavenging activity of the CPAE was often evaluated according to its IC50; the lowest IC50 value corresponds to the highest free radical scavenging activity. It is obvious that CPAE had DPPH, NO, FRAP, and OH free radical scavenging activities. The CPAE antioxidants properties were lower than those of ascorbic acid which was used as a positive control, at *p* < 0.05 (Table 3).

### 3.3. Anti-Digestive Enzymes Effect of CPAE

**Figure 2:**
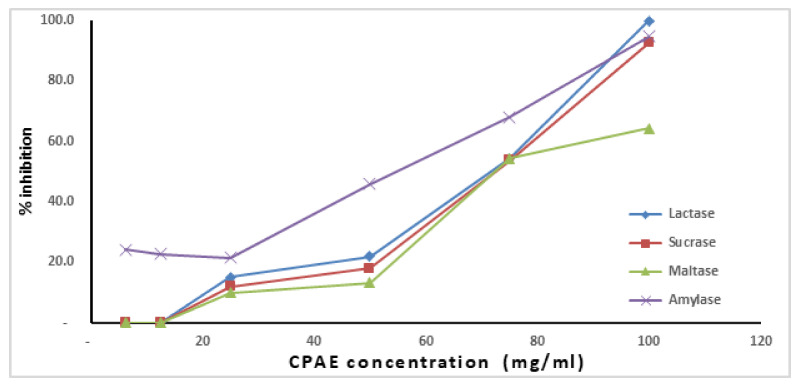
Anti-digestive enzyme effect of carob pod aqueous extract (CPAE).

The results in Figure 2 revealed that Amylase, Maltase, Sucrase, and Lactase activities were strongly inhibited by CPAE in a concentration-dependent manner.

### 3.4. The Hemolytic Effect of CPAE

**Figure 3:**
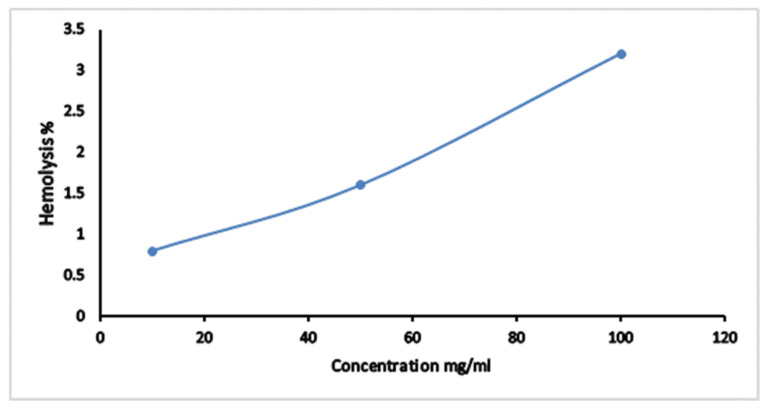
The antihemolytic activity of carob pod aqueous extract (CPAE).

Hemolytic activity of CPAE was screened against normal human erythrocytes. Figure 3 showed that the hemolytic percent was increased from 0.7 to 3.2% when the concentration of CPAE increased in the range from 10 mg/mL to 100 mg/mL. The calculated hemolytic IC50 was 1562.5 mg/mL.

### 3.5. Anti-Microbial Activity of CPAE

#### 3.5.1. The Antibacterial Activity against Gram-Negative, Gram-Positive Bacteria and Antifungal Effect

Table 4 shows that the CPAE had a significant antibacterial property against *Bacillus subtilis*, *Staph. aureus*, and *Escherichia coli*. However, CPAE did not show any activity against Gram-negative bacteria *Pseudomonas aeruginosa*. Moreover, it exhibited a potent antibacterial effect compared to that of gentamicin (positive control antibiotic) in the case of *Staphylococcus aureus* and *Escherichia coli* with ZOI (25 and 24 mm, respectively). In addition, the extract exhibited an antifungal effect against *Candida albicans*, while it did not show any activity against *Aspergillus falvus* Table 4.

#### 3.5.2. Antiviral Activity of CPAE

Table 5 indicated that the CPAE was considered safe for Vero cells until 5000 µg/mL where cell viability was 96.5%. Moreover, our results showed that CPAE inhibited HSV-1 in a concentration-dependent manner (Table 6).

## 4. Discussion

The phytochemical investigation of CPAE in the current study revealed the presence of flavonoids, total polyphenols, alkaloids, carbohydrates, and amino acids. The presence of these phytochemicals marked it understandable why carob pods are a prominent medicinal plant for the treatment of various diseases. In agreement with this finding, Ayache et al. (2020) demonstrated that carob pulp contains a significant amount of phenolic compounds and flavonoids (1.8 EAG/100 gDW, 0.6 EC/100 gDW) respectively [28]. In addition, Nawel et al. (2018) found that carob pods contain high content of alkaloids 56.51 ± 1.02 mg/100 g [29]. Moreover, there are many reports demonstrating the presence of alkaloids in carob [30,31] which can explain the traditional therapeutic uses of carob. Moreover, the high content of amino acids increases the nutritional value of carob; according to the World Health Organization (WHO), carob has all the essential amino acids [32]. In addition, the HPLC analysis revealed that CPAE contained a high content of gallic acid, catechin, and protocatechuic acid, respectively, and gallic acid is the most abundant phenolic compound in CPAE. These results were in agreement with previous reports, which revealed that gallic acid was the major phenolic acid present in carob pods [30,33]. Many studies have demonstrated that these molecules are well known for their antioxidant and anticancer properties [32,34]. There are several data about the component of carob performed by HPLC, Ritib et al. 2015 found that the main components of CPAE are pyrogallol 48.0% and catechin 19.1%, and the gallic acid is present in a small content 3.1% [35]. In addition, the study by Ghanemi et al. (2017) showed that m-coumaric acid and gallic acid are the major polyphenols present in carob (2192.3 and 1445.3 µg/g DW, respectively) [36]. Moreover, Goulas and Georgiou found that the main components of water carob pod extract are myricetin 94.5%, gallic acid 80%, epicatechin 77.3%, and rutin 67% [37]. Another study by Al-Olayan et al. (2016) found that carob pods contain high content of polyphenols 68.3 ± 5.8 mg gallic acid equivalent/g dried pod of polyphenols and a relatively low amount of flavonoids 9.8 ± 2.2 mg quercetin equivalent/g dried pod. The main components of CPAE are kaempferol (53.53%) and gallic acid (11.19%) [30]. This variation in phenolic content depends on many factors such as genetic, geographical, environmental, physiological, and cultural conditions, preparation of samples, extraction, and method of analysis [38]. Due to the complex nature of phytochemicals present in carob pods, we used multiple assays to provide preliminary evidence about the antioxidant activity of the Egyptian carob pods. Firstly, our results of in vitro antioxidant activity indicated that the CPAE had a strong ability to inhibit DPPH, and this activity is due to the presence of active constituents in carob pods which have the ability to donate hydrogen to a free radical in order to remove the odd electron which is responsible for the radical’s reactivity. Our results were in agreement with previous findings, which recorded that carob had a strong DPPH scavenging activity [28,34,39]. Further, the reducing power of CPAE was evaluated with its ability to transform Fe^3+^ to Fe^2+^ through electron transferability, which acts as a significant indicator of its antioxidant activity. Hence, iron participates in many cellular functions, and any excess iron can generate reactive oxygen species through the Fenton reaction. The iron-chelating capacity would prevent transition metals from participating within the commencement of oxidative stress [40]. Here, the Fe^2+^ chelating activity of the CPAE was increased with increasing concentration, reaching a level of 92.79% at high concentrations (1 g/mL). These results were in agreement with many other studies that reported the ability of carob to reduce Fe3+ to Fe2+could be due to the presence of polyphenols [28,39]. In agreement with these findings, Goulas and Georgiou found that *ceratonia siliqua* extract has a good antioxidant activity as evidenced by the DPPH IC50 (1.9 mg/mL) and FRAP (339.7 mg FeSO4 100 g^−1^) [37]. In another study by Abidar et al. (2020) found that carob has a strong antioxidant activity with a DPPH IC50 = 0.116 mg/mL and a high FRAP with an EC50 = 0.123 mg/mL [41].

In addition, the results of this study indicated that CPAE was an effective hydroxyl radical scavenger with IC50 = 7.07 mg/mL, and the result was in line with the findings of Nadia Amessis et al. (2016), who reported that *ceratonia siliqua* had a hydroxyl radical scavenging activity of 38% [42]. The hydroxyl radicals are formed as a result of the reduction of oxygen to water and are considered the major active oxygen species implicated in lipid peroxidation and many biological damages leading to many diseases such as atherosclerosis, cancer, and neurological disorders, and can be prevented by the action of non-reducing substances [43]. Additionally, nitric oxide plays a significant role in the pathogenesis of various diseases associated with inflammation [44]. As shown in our results, CPAE produced significant antioxidant effects against nitric oxide production at serial dilutions; this result is consistent with Rico et al. (2019) who reported that carob pods significantly inhibit NO production [45]. These observations give an indication of the strong antioxidant potential of the CPAE, which is confirmed with its action on DPPH, FRAP, OH, and NO.

Phenolic compounds as depicted in carob pods are good electron donors and could terminate the radical chain reaction by converting free radicals into more stable products [28]. The antioxidant activity of phenolics depends on the number and substitution of the hydroxyl group. As such, carob’s antioxidant activity can thus be attributed to the presence of gallic acid, protocatechuic, catechin, p-hydroxybenzoic, and vanillic acid [46].

There are several methods to control blood glucose level; one of them is the control of postprandial hyperglycemia via delaying the absorption of carbohydrates after food intake [47]. This could be done through the inhibition of α-amylase and α-glucosidase enzymes (maltase, lactase, and sucrase) present in the gastrointestinal tract [48]. Inhibitors of these enzymes slow down the digestion of carbohydrates, causing a decrease in glucose absorption rate, thereby reducing the increase in blood sugar after a meal [49]. Although carob contains a high content of carbohydrates, it exhibited a significant antidiabetic effect, which gave carob great importance as a natural sweetener for diabetics. As presented in Figure 1, our results indicated that CPAE had a potent inhibitory effect against all digestive enzymes. These findings were in agreement with Custódio et al. (2014) who confirmed that the leaves and stem bark of the carob tree has a high inhibitory effect on the activity of α-amylase, α-glucosidase, and also display significant antioxidant activity [50]. Many studies have reported that polyphenols, mainly flavonoids, phenolic acids, and tannins, have a significant benefit in inhibiting α-glucosidase and α-amylase, which are the two critical enzymes involved in the conversion of dietary carbohydrates to glucose. [51].

The results of this study also showed that CPAE had a significant antibacterial effect against pathogenic gram-positive and gram-negative bacteria (*Bacillus subtilis*, *Staphylococcus aureus*, and *Escherichia coli*). These results were in agreement with Hsouna et al. (2015), who reported that carob ethyl acetate extract showed antimicrobial activity against many pathogenic bacteria [52]. The antibacterial activity of CPAE could be explained by the presence of biologically active compounds such as phenolic acids and flavonoids that impact the growth and metabolism of microorganisms. The phenols and flavonoids contribute significantly to the antibacterial activity and can form complexes with the cell wall and also disrupt bacterial envelopes [53]. Further, Tassou et al. (1997) reported that carob had an inhibitory effect against many pathogenic bacteria [54]. CPAE is very rich in gallic acid, which can inhibit the motility, adherence, and biofilm formation of many pathogenic bacteria [55]. Gallic acid can also disrupt the integrity of the cell membrane in Gram-positive and Gram-negative bacteria and change the charge, hydrophobicity, and permeability of the membrane surface [56]. Furthermore, protocatechuic has broad-spectrum antibacterial activity against a wide range of bacterial species [57].

In addition, The CPAE exhibited a significant inhibitory effect on the growth of *Candida albicans,* suggesting that the studied CPAE are possibly a secure and natural source of antifungal agents. Hussein et al. (2011) reported that carob leaves had a significant antibacterial and fungal effect [58]. In addition, our results indicate that CPAE exhibited a safe effect on Vero cells and revealed a significant inhibitory effect against HSV-1. As a result of the highest content of gallic acid in CPAE, which explains the antiviral effect of HSV-1, Kratz et al. (2008) reported that gallic acid has the ability to inhibit the herpes simplex virus’ (HSV-1 and HSV-2) attachment and penetration [59].

## 5. Conclusions

This study indicated that CPAE contained phenolic, flavonoid, alkaloid, and amino acids, and carbohydrates. Thus, CPAE possessed antioxidants activity. In addition, it had hypoglycemic effect via inhibiting the intestinal enzymes, maltase, sucrase, lactase, and amylase. Furthermore, it had strong antimicrobial activity against *Staph. aureus*, *Escherichia coli*, *Escherichia coli* and *HSV-1*. Therefore, this study suggested that CAPE can be used as a food supplement and supportive therapeutic agent for microbial and diabetic treatment.

## Figures and Tables

**Table 1:** Quantitative phytochemical component of the carob pod aqueous extract.

Parameters	Concentration
Total flavonoids (Rutin/100 mg CPAE)	0.752 ± 0.11
Total phenolics (GA/100 mg CPAE)	0.348 ± 0.07
Total amino acids (mg/mL)	43.28 ± 2.1
Total alkaloids (mg/mL)	15.3 ± 0.98
Total carbohydrates (mg/mL)	5.1 ± 0.23

The value expressed mean ± SE, Gallic acid, GA; carob pod aqueous extract, CPAE.

**Table 2:** The constituents of carob pod aqueous extract.

Compound	Concentration (µg/g)	Retention Time
Gallic acid	833.751	3.74
Catechin	180.197	11.67
Protocatechuic acid	110.576	7.98
Cinnamic acid	23.335	39.7
*p*-coumaric acid	12.707	36.42
Rutin	10.983	35.9
Gentisic acid	3.487	7.04
*p*-hydroxybenzoic acid	2.731	9.88
Vanillic acid	2.261	17.47
Ferulic acid	2.038	20.79

**Table 3:** IC50 of carob pod aqueous extract and ascorbic acid as positive control antioxidants activities.

Parameters	CPAE IC50 (mg/mL)	Ascorbic Acid IC50 (mg/mL)
DPPH scavenging activity	3.78 ± 0.08 *	1.59 ± 0.01
Ferric reducing activity	6.30 ± 0.11 *	4.21 ± 0.25
NO scavenging activity	4.33 ± 0.06 *	3.48 ± 0.31
•OH, scavenging activity	7.07 ± 0.12 *	1.32 ± 0.01

In each raw, CPAE mean with * was significantly difference compared to that of the ascorbic acid mean, at *p* < 0.05.

**Table 4:** Antimicrobial activity of carob pod aqueous extract.

Pathogenic Microorganism	Inhibition Zoon (mm)	
	CPAE	Postive Control
*Bacillus subtilis* (ATCC 6051)	17.0 ± 0.12 *	25.0 ± 0.3
*Staph. aureus* (ATCC 6538)	25.0 ± 0.2 *	15.0 ± 0.11
*Escherichia coli* (ATCC 8739)	24.0 ± 0.3 *	17.0 ± 0.09
*Pseudomonas aeruginosa* (ATCC 90274)	N.A	22.0 ± 0.12
*Candida albicans* (ATCC 10221)	15 ± 0.13	N.A
*Aspergillus falvus* (ATCC 9643)	N.A	N.A

In each raw, CPAE mean with * was significantly difference compared to that of gentamicin mean, at *p* < 0.05. N.A: No activity, positive control is gentamicin, Carob pod aqueous extract, CPAE.

**Table 5:** Effect of carob pod aqueous extract on Vero cells viability.

Concentration (µg/mL)	Viability %
10,000	39.4 ± 0.01 ^a^
5000	96.5 ± 0.05 ^b^
2500	97.4 ± 0.02 ^b^
1250	99.0 ± 0.04 ^c^
625	99.8 ± 0.05 ^c^
312.5	100.0 ± 0.08 ^c^
IC50	9154.22

The value expressed mean ± SE. Means with different superscript letters were significantly different at *p* < 0.05. Mean with letter (a) was the lowest one while mean with letter (c) was the highest one.

**Table 6:** Effect of carob pod aqueous extract on the viability of Vero cells infected with HSV-1.

μg/mL	Viability	Antiviral Effect %
0	37.94	0.00
1250	40.78	4.58 ± 0.01 ^a^
2500	50.40	20.08 ± 0.25 ^b^
5000	66.80	46.50 ± 1.5 ^c^

Value expressed mean ± SE. Means with different superscript letters were significantly different at *p* < 0.05. Mean with letter (a) was the lowest one while mean with letter (c) was the highest one.

## Data Availability

The data presented in this study are available on request from the corresponding author.
